# Propensity Score-Matched Analysis of the Survival Benefit from Kidney Transplantation in Patients with End-Stage Renal Disease

**DOI:** 10.3390/jcm7110388

**Published:** 2018-10-26

**Authors:** Ming-Ju Wu, Tung-Min Yu, Cheng-Li Lin, Chia-Hung Kao

**Affiliations:** 1 Division of Nephrology, Department of Medicine, Taichung Veterans General Hospital, Taichung 400, Taiwan; wmj530@gmail.com (M.-J.W.); yu5523@gmail.com (T.-M.Y.); 2 Graduate Institute of Biomedical Sciences and School of Medicine, China Medical University, Taichung 404, Taiwan; 3 Graduate Institute of Biomedical Sciences, College of Life Science, National Chung Hsing University, Taichung 404, Taiwan; 4 School of Medicine, Chung-Shan Medical University, Taichung 404, Taiwan; 5 Management Office for Health Data, China Medical University Hospital, Taichung 404, Taiwan; orangechengli@gmail.com; 6 College of Medicine, China Medical University, Taichung 404, Taiwan; 7 Department of Nuclear Medicine and PET Center, China Medical University Hospital, Taichung 404, Taiwan; 8 Department of Bioinformatics and Medical Engineering, Asia University, Taichung 404, Taiwan

**Keywords:** dialysis, kidney transplantation, propensity score matching, survival

## Abstract

Background: Several comparison studies have suggested that kidney transplantation (KT) could reduce mortality in patients with end-stage renal disease (ESRD). Selection criteria bias is common in the selection of dialysis patients for control groups. In this study, we compared the survival outcome between KT recipients and comparable propensity score-matched dialysis patients. Methods: We used Taiwan’s National Health Insurance Research Database to identify patients newly diagnosed with ESRD between 2000 and 2010. We separated them into two groups: a KT group and non-KT dialysis-only group. To evaluate the survival outcome, we compared each patient with KT to a patient on dialysis without KT using propensity score matching. Results: In total, 1276 KT recipients and 1276 propensity score-matched dialysis patients were identified. Compared with the propensity score-matched dialysis patients, the patients who underwent KT exhibited significantly higher 5-year and 10-year survival rates (88% vs. 92% and 74% vs. 87%, both *p* < 0.05). The crude and adjusted hazard ratios for mortality were 0.55 and 0.52 in patients with KT (both *p* < 0.001). Mortality was insignificantly higher for patients who were on dialysis for longer than 1 year prior to KT compared with those on dialysis for less than 1 year. Conclusion: This study used a propensity score-matched cohort to confirm that KT is associated with lower risk of mortality than dialysis alone in patients newly diagnosed with ESRD.

## 1. Introduction

Because populations are aging and diabetes mellitus has become more prevalent, the incidence of end-stage renal disease (ESRD) has increased worldwide. ESRD often causes mortality and disability and thus represents a growing public health problem [1]. Consequently, the use of renal replacement therapies has risen globally. Among the three renal replacement therapies, kidney transplantation results in significantly higher quality of life and reduced mortality than hemodialysis and peritoneal dialysis [2,3,4,5,6]. However, most of the survival data available are observational and thus negatively affected by confounding factors and limitations. The effect of selection bias on survival analysis, especially with respect to age and comorbidities, for patients with ESRD who do and do not undergo kidney transplantation remains unclear [7,8]. Our previous study found that kidney transplantation increases survival among patients with lupus nephritis and ESRD compared with hemodialysis and peritoneal dialysis [9]. Besides age, several comorbidities were seen with inconsistent frequency among patients undergoing kidney transplantation, peritoneal dialysis, and hemodialysis in that study. Similar differences in the baseline characteristics of the study cohort appear in a report from Tennankore and colleagues [10]. Recent studies have found a marked reduction over the 2010s in mortality rate among patients receiving maintenance dialysis [11,12]. Data from ESRD registries in the United States, Europe, and Australasia have shown a modest improvement in mortality rate for people with dialysis-treated ESRD, but it is unclear whether the magnitude of this improvement is comparable with that observed in ESRD patients who receive kidney transplants [13,14,15].

Because most kidney transplant recipients are younger and have fewer comorbidities compared with the dialysis population, the survival rate between all dialysis patients and kidney transplant recipients cannot be directly compared. However, ethical issues preclude the performance of randomized control trials comparing dialysis patients and kidney transplant recipients. Those on transplant waiting lists may be better candidates for inclusion in an investigative study into the difference between the survival rates of dialysis and kidney transplant patients. To further limit the effect of biases caused by confounding variables, we consider forming an adjusted cohort through propensity score matching, as well as implementing equal age and sex categories, to be the best study design for clearly identifying the difference between the survival outcome of each renal replacement therapy. Although it cannot replace a truly randomized trial, propensity score matching is a powerful tool for adjusting confounding variables and reducing the number of treatment selection biases [16,17]. In the current study, we used an adjusted waitlist cohort to evaluate the incidence rate and hazard ratio of mortality in patients with ESRD with or without kidney transplants.

## 2. Materials and Methods

### 2.1. Data Source

We conducted a retrospective nationwide cohort study by analyzing the Registry for Catastrophic Illness Patients Database (RCIPD) of the National Health Insurance (NHI) program in Taiwan. The NHI program, which covers 99% of the 23 million inhabitants of Taiwan, was launched on 1 March 1995. The RCIPD contains the medical records for each patient diagnosed with a catastrophic illness from 1996 to 2011. Being listed in the RCIPD exempts patients, including dialysis patients, from copayments for the medical services related to their conditions. The Research Ethics Committee of China Medical University and Hospital in Taiwan approved this study (CMUH104-REC2-115-CR3). 

### 2.2. Study Enrollees

We identified patients first listed in the RCIPD from 2000 to 2010 with catastrophic illness registration cards for ESRD who underwent regular dialysis (ICD-9-CM Code 585) and were evaluated for kidney transplantation. We separated these patients into two groups: a kidney transplantation group (ICD-9-CM Code V42.0) and a comparison group of patients receiving dialysis but not transplants. The study index date was defined as the date on which a kidney transplant recipient underwent the transplant procedure, and the corresponding comparison dialysis patients were followed from a randomly assigned date in the same year. Patients diagnosed with cancer (ICD-9-CM Codes 140–208) and those younger than 18 years old or with missing demographic data were also excluded. For each kidney transplant patient, a dialysis patient who did not receive a transplant was selected for comparison; the dialysis-only patients were selected and frequency matched according to age (in 5-year increments), sex, and year of index date on the basis of the same exclusion criteria. A second method of comparison matched the kidney transplantation group and dialysis group with propensity scores at a 1:1 ratio to minimize selection bias. Propensity scores were calculated using logistic regression to estimate the probability of receiving a kidney transplant based on the baseline variables of age, sex, index date, and comorbidities (hypertension, hyperlipidemia, diabetes, coronary artery disease, atrial fibrillation, heart disease (ICD-9-CM Codes 420–429), hepatitis B virus, hepatitis C virus, and peripheral vessel disease). All patients were followed until death, withdrawal from the NHI program, or 31 December 2011 (whichever occurred first). The cumulative censoring rate over 12 years was 7.55% in the kidney transplantation cohort, which was slightly lower than that in the comparison cohort (13.1%). The possible reasons for the discontinuity of national health insurance include death, withdrawal of insurance, immigration, prison sentence, etc.

### 2.3. Statistical Analysis

Demographic characteristics and the prevalence of comorbidities were compared between the groups using the chi-square test for categorical variables and a two-sample *t* test for the continuous variables in the age- and sex-matched groups. A standardized difference of ≤0.10 indicates a negligible difference between the two propensity score-matched cohorts. The cumulative incidence of mortality was calculated using the Kaplan–Meier method, and the log-rank test was used to examine the differences between the two groups that were propensity score matched. We calculated the incidence density of mortality according to person-years in each group. Univariable and multivariable Cox proportional-hazard regression models were used to determine the effect of kidney transplantation on the risk of mortality by using a hazard ratio (HR) and its 95% confidence interval (CI). The multivariable models were simultaneously adjusted for age, sex, and the existence of hypertension, hyperlipidemia, diabetes, coronary artery disease, atrial fibrillation, heart disease, hepatitis B virus, hepatitis C virus, and peripheral vessel disease. Based on propensity score matching, Cox proportional hazards models stratifying on the matched pairs were performed to estimate the hazard ratio (HR) and 95% confidence intervals (CI) of developing mortality associated with kidney transplantation, compared with the comparison cohort. Patients were stratified according to age, sex, comorbidity, and follow-up duration, and the relative risk of mortality in the kidney transplantation group compared with the dialysis-only group was analyzed using Cox models. We conducted further data analysis to assess the effect of the dialysis treatment period before kidney transplantation on the risk of mortality, comparing patients receiving dialysis for less than 1 year and for more than 1 year. We also compared the risk of mortality between two groups using different propensity score methods for sensitivity analysis. All analyses were conducted using SAS software version 9.4 (SAS Institute, Cary, NC, USA), and the two-tailed statistical significance level was set at *p* < 0.05.

## 3. Results

**Figure 1:**
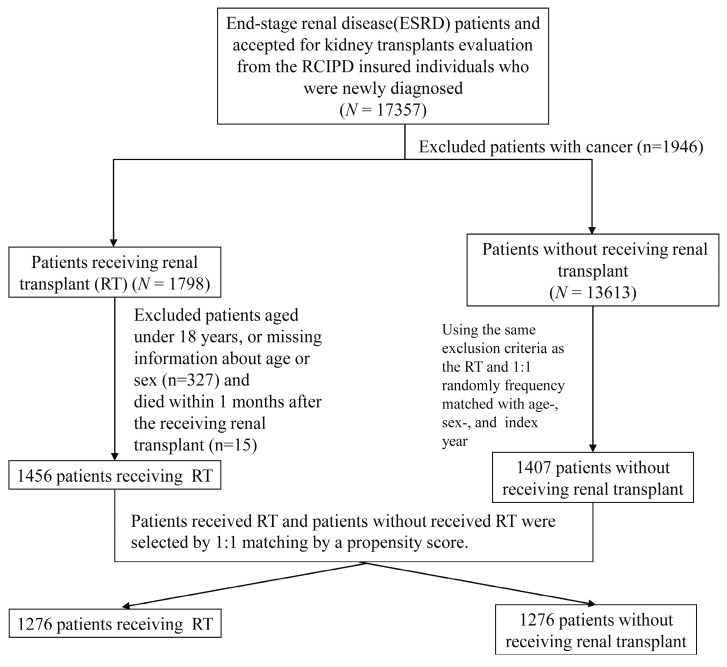
Patient enrollment procedure.

**Figure 2:**
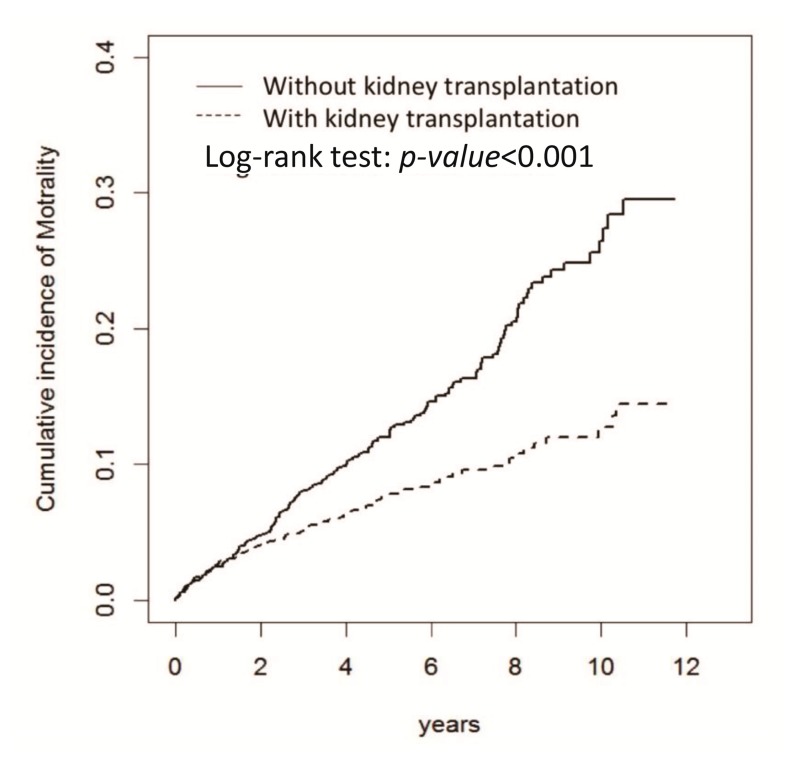
Survival curves comparing patients with end-stage renal disease in the propensity score-matched cohorts who received a kidney transplant or only received dialysis.

Figure 1 shows the patient enrollment process for the study, which selected 17,357 newly diagnosed patients with ESRD. This study evaluated 1456 patients in the kidney transplantation group and 1407 patients in the dialysis-only group; the patients in the two groups were matched by age and sex (Table 1). In addition, 1276 patients in the kidney transplantation group were matched with 1276 dialysis-only patients using propensity score matching. The patients in the groups matched by age and sex were predominately between the ages 35 and 49 years (approximately 46–48%) and were mostly female (approximately 52%); however, the patients in the kidney transplantation group were significantly more likely to exhibit the comorbidities of hypertension and coronary artery disease. The kidney transplantation group presented significantly less prevalent heart disease. After propensity score matching, age, sex, and comorbidity were more similarly distributed in the two cohorts (Table 1). The mean age in the kidney transplantation group was 43.5 ± 11.1 years, and the mean age was 43.3 ± 10.6 years in the dialysis-only group. Figure 2 presents the cumulative incidence of mortality over 12 years of follow-up for the propensity score-matched groups. The cumulative incidence of mortality was significantly lower for the kidney transplantation cohort than the dialysis-only cohort (log-rank test: *p* < 0.001). The 5-year patient survival rate was 92% in the kidney transplantation group and 88% in the dialysis-only group, and the 10-year patient survival rate was 87% in the kidney transplantation group and 74% in the dialysis-only group (both *p* < 0.05). 

The mean follow-up periods for the age- and sex-matched patients who died during the study period in the kidney transplantation group and the dialysis-only group were 5.31 ± 3.07 and 5.02 ± 2.96 years, respectively (Table 2). The overall incidence density for mortality was lower in the kidney transplant patients than in the dialysis controls (1.46 vs. 2.93 per 1000 person-years), with an adjusted HR of 0.51 (95% CI = 0.41–0.64). The risk of mortality was significantly lower in the kidney transplantation group (41%) than in the dialysis group according to the propensity score (HR = 0.59, 95% CI = 0.44–0.78). 

The survival benefit for kidney transplant recipients among the patients with ESRD and with different ages, sexes, follow-up durations, and comorbidities was consistent, except in those patients with peripheral vessel disease and atrial fibrillation (Table 3). The relative risk of mortality for the age-specific kidney transplant recipients compared with the dialysis group was not significantly lower across all age groups. When stratified by sex, patients who received kidney transplants had a significantly lower risk of mortality compared with patients who did not receive kidney transplants among both the women (HR = 0.58, 95% CI = 0.36–0.95) and men (HR = 0.64, 95% CI = 0.42–0.99). Among the patients without particular comorbidities, kidney transplant patients had a lower risk of mortality than the dialysis-only group (HR = 0.63 for patients without hyperlipidemia; HR = 0.62 for patients without diabetes; HR = 0.66 for patients without coronary artery disease; HR = 0.58 for patients without atrial fibrillation; HR = 0.58 for patients without heart disease; HR = 0.64 for patients without hepatitis B virus; HR = 0.64 for patients without hepatitis C virus; HR = 0.58 for patients without peripheral vessel disease). The HR of mortality decreased as the follow-up duration lengthened among the kidney transplant recipients compared with the dialysis patients (HR = 0.31 with 95% CI = 0.14–0.68 for dialysis period >2 and ≤3 years; HR = 0.23 with 95% CI = 0.10–0.56 for >5 years). 

We next assessed the effect of the duration of pretransplant dialysis on the risk of mortality in all propensity score-matched kidney transplant patients (Table 4). Compared with the kidney transplant recipients receiving dialysis for less than 1 year, the kidney transplant recipients receiving dialysis for a longer pretransplant period had an insignificantly higher adjusted HR (adjusted HR = 1.07 with 95% CI = 0.40–2.83 for dialysis durations of >1 and ≤3 years; adjusted HR = 1.39 with 95% CI = 0.53–3.62 for dialysis duration >3 and ≤5 years; adjusted HR = 1.41 with 95% CI = 0.53–3.77 for >5 and ≤7 years; adjusted HR = 1.65 with 95% CI = 0.62–4.36 for >7 years; all *p* > 0.05). 

Similar results were observed for mortality through different propensity score methods; the kidney transplant cohort had a lower risk of mortality than the comparison cohort (Table 5).

## 4. Discussion

This 12-year follow-up study is the first large-scale propensity score-matching investigation of the survival benefit of kidney transplantation for patients newly diagnosed with ESRD and undergoing dialysis in Taiwan, a region with a very high prevalence of ESRD. We made three major findings. First, we demonstrated a significantly lower risk of mortality (adjusted HR = 0.52 with 95% CI = 0.43–0.70) for patients with ESRD who received a kidney transplant compared to those who did not. In 2008, Huang CC and colleagues reported overall 10-year patient survival rates of 35% for peritoneal dialysis patients and 33.8% for hemodialysis patients [18]. However, the survival rate for dialysis patients in our propensity score-matched cohort was much higher, 74%. In fact, dialysis patients in our study cohort were younger and had fewer comorbidities. In other words, this finding suggests that younger dialysis patients without comorbidities could have a favorable long-term survival rate. However, patients who received a kidney transplant had a significantly higher 10-year survival rate—13% higher, 87% vs. 74%—than the propensity score-matched dialysis patients.

Second, our findings confirmed that selection biases affected earlier survival analysis studies. Previous Taiwanese national data reported by Wu PH and colleagues indicated a mean age of 60.96 ± 13.92 years among 79,645 dialysis patients and that 51.2% of these patients were diabetic [19]. The mean age in our matched cohort study was only 43.5 years, and the percentage of patients with diabetes mellitus was approximately 12.5%. Moreover, we confirmed a remarkable difference in the prevalence of various comorbidities between patients with ESRD who received kidney transplants or not, even after matching for age and sex (Table 1). Before propensity score matching, more dialysis patients without a kidney transplant had coronary artery disease, heart disease, and hypertension. These findings are consistent with clinical practice; cardiovascular events are the primary cause of death in both kidney transplant recipients and dialysis patients [20,21]. The risk of a cardiovascular event is a major concern during evaluations of patients with ESRD for kidney transplant, and clinicians may discourage dialysis patients with comorbidities from taking the risk of kidney transplantation surgery [22]. Therefore, a younger age and fewer, or less severe, comorbidities may have contributed to a higher survival rate in our propensity score-matched cohort. In summary, the results of this propensity score-matching cohort study confirm that kidney transplantation provides a significantly better survival outcome for dialysis patients with similar ages and comorbidities. 

We also determined the HRs of mortality in dialysis populations across different age ranges, sexes, comorbidities, and follow-up durations (Table 3). The survival benefit of kidney transplantation was consistent in patients with various comorbidities, with peripheral vessel disease as the exception. The adjusted HR was significantly lower in patients with a follow-up duration longer than 2 years; thus, patients receiving dialysis for a longer period of time, even longer than 5 years, still had a higher survival rate after kidney transplantation. Our findings could help clinicians focus on receiving the necessary information in patient surveys during kidney transplantation evaluation.

Studies have found that a lengthier duration of pretransplant dialysis was an independent risk factor for patient death after kidney transplantation [23,24,25]. The survival rate for dialysis patients has improved in the past decade, and it would be interesting to determine whether a longer duration of dialysis before receiving a kidney transplant continues to be associated with a worse survival duration after kidney transplantation. We were surprised that our findings failed to support the negative effect of a longer waiting time before kidney transplantation on patient survival. Although the adjusted HR increased gradually as the waiting time before kidney transplantation increased, the difference was not significant. We propose that the different baseline characteristics and improved survival of dialysis patients in the past decade may have been the cause. 

The cumulative regular dialysis population in Taiwan was more than 80,000 at the end of 2017. The transplant rate is relatively low in Taiwan. According to the online data from the Taiwan Organ Registry and Sharing Center, less than 10% regular dialysis patients are on the waiting list for kidney transplantation. Moreover, the indication for the waiting list for cadaveric kidney transplantation, such as age and comorbidities, widely varies between different transplant centers. Thus, we suggest that matching from the waitlist could be more difficult than propensity score-matching a dialysis population.

The data compiled in the RCIPD limited what could be done in our study. The dataset does not distinguish between live and cadaveric kidney transplantation, but a live kidney transplantation results in a considerably higher long-term survival rate than a cadaveric kidney transplantation. Additionally, we were unable to assess the effect of the condition of the donor kidney. Using a kidney from a donor who has diabetes mellitus, is geriatric, or has viral hepatitis is no longer contraindicated for kidney transplantation. The waiting list criteria for kidney transplantation have also changed over the past few decades [26,27,28,29], and preemptive kidney transplantation is associated with optimal outcomes [30]; however, preemptive kidney transplantation recipients were not enrolled in this study. 

## 5. Conclusions

This study confirms the survival benefit of kidney transplantation in patients with ESRD. In light of the survival data presented, we conclude that it is reasonable to perform kidney transplantation on all dialysis patients without contraindication.

## Figures and Tables

**Table 1:** Demographic characteristics and comorbidities in kidney transplant and dialysis-only cohorts; age- and sex-matched and propensity score-matched groups.

	Age and Gender Matched			Propensity Score Matched		
Variable	Kidney Transplantation		*p*-Value	Kidney Transplantation		Standardized Differences
	No	Yes		No	Yes	
	*N* = 1407	*N* = 1456		*N* = 1276	*N* = 1276	
Age, year			0.34			
≤34	319 (22.7)	364 (25.0)		293 (23.0)	310 (24.3)	0.03
35–49	668 (47.5)	670 (46.0)		618 (48.4)	578 (45.3)	0.06
≥50	420 (29.9)	422 (29.0)		365 (28.6)	388 (30.4)	0.04
Mean ± SD †	43.6 (10.7)	43.1 (11.0)	0.25	43.3 (10.6)	43.5 (11.1)	0.01
Sex			0.96			
Female	727 (51.7)	751 (51.6)		648 (50.8)	649 (50.9)	0.002
Male	680 (48.3)	705 (48.4)		628 (49.2)	627 (49.1)	0.002
Comorbidity						
Hypertension	1145 (81.4)	1252 (86.0)	<0.001	1060 (83.1)	1072 (84.0)	0.025
Hyperlipidemia	433 (30.8)	462 (31.7)	0.58	373 (29.2)	390 (30.6)	0.029
Diabetes	178 (12.7)	182 (12.5)	0.9.00	152 (11.9)	160 (12.5)	0.019
Coronary artery disease	410 (29.1)	298 (20.5)	<0.001	306 (24.0)	298 (23.4)	0.015
Atrial fibrillation	21 (1.49)	17 (1.17)	0.45	20 (1.57)	17 (1.33)	0.020
Heart disease	469 (33.3)	391 (26.9)	<0.001	380 (29.8)	389 (30.5)	0.015
Hepatitis B	105 (7.46)	121 (8.31)	0.40	99 (7.76)	107 (8.39)	0.023
Hepatitis C	98 (6.97)	106 (7.28)	0.74	85 (6.66)	92 (7.21)	0.022
Peripheral vessel disease	33 (2.35)	26 (1.79)	0.29	24 (1.88)	26 (2.04)	0.022

Chi-square test; †: *t* test. §A standardized difference of ≤0.10 indicates a negligible difference between the two cohorts.

**Table 2:** Incidence and hazard ratio of mortality in patients receiving a kidney transplant compared with dialysis-only patients by match type between study cohorts.

	Age and Gender Matched		Propensity Score Matched	
	Kidney Transplantation		Kidney Transplantation	
	No	Yes	No	Yes
	(*N* = 1407)	(*N* = 1456)	(*N* = 1276)	(*N* = 1276)
Person-years	7060	7733	6388	6561
Follow-up time (years, mean ± SD)	5.02 ± 2.96	5.31 ± 3.07	5.01 ± 2.94	5.14 ± 3.04
Mortality				
Event	207	113	179	101
Rate #	2.93	1.46	2.80	1.54
cHR (95% CI)	1 (Reference)	0.50 (0.40, 0.63) ***	1 (Reference)	0.59 (0.44, 0.78) ***
aHR † (95% CI)	1 (Reference)	0.51 (0.41, 0.64) ***		

Rate #, incidence rate per 100 person-years. cHR, crude hazard ratio; aHR, adjusted hazard ratio. aHR †: multivariable analysis including age, sex, hypertension, hyperlipidemia, diabetes, coronary artery disease, atrial fibrillation, heart disease, hepatitis B, hepatitis C, and peripheral vessel disease. *** *p* < 0.001.

**Table 3:** Incidence (per 100 person-years) and hazard ratio of mortality in patients receiving a kidney transplant compared with dialysis-only patients matched by age, sex, comorbidity, and follow-up duration.

	Propensity Score Matched						
Variables	Event	PY	Rate #	Event	PY	Rate #	HR (95% CI)
Kidney Transplantation							
	No			Yes			
Age, years							
≤34	33	1645	2.01	18	1815	0.99	0.67 (0.30, 1.48)
35–49	77	3310	2.33	44	3185	1.38	0.58 (0.33, 1.04)
≥50	69	1433	4.82	39	1560	2.50	0.64 (0.33, 1.24)
Sex							
Female	80	3427	2.33	44	3474	1.27	0.58 (0.36, 0.95) *
Male	99	2960	3.34	57	3087	1.85	0.64 (0.42, 0.99) *
Comorbid							
Hypertension							
No	21	1332	1.58	18	1263	1.42	0.87 (0.41, 1.82)
Yes	158	5055	3.13	83	5298	1.57	0.49 (0.36, 0.68) ***
Hyperlipidemia							
No	122	4802	2.54	69	4866	1.42	0.63 (0.44, 0.91) *
Yes	57	1585	3.60	32	1695	1.89	0.55 (0.31, 0.97) *
Diabetes							
No	130	5796	2.24	81	5944	1.36	0.62 (0.44, 0.88) **
Yes	49	592	8.28	20	617	3.24	0.06 (0.01, 0.42) **
Coronary artery disease							
No	108	4999	2.16	69	5185	1.33	0.66 (0.46, 0.93) *
Yes	71	1389	5.11	32	1376	2.33	0.36 (0.21, 0.64) ***
Atrial fibrillation							
No	175	6312	2.77	98	6485	1.51	0.58 (0.44, 0.78) ***
Yes	4	75	5.31	3	76	3.96	-
Heart disease							
No	112	4727	2.37	59	4774	1.24	0.58 (0.41, 0.84) **
Yes	67	1660	4.04	42	1787	2.35	0.69 (0.43, 1.11)
Hepatitis B							
No	162	6037	2.68	92	6138	1.50	0.64 (0.47, 0.86) **
Yes	17	351	4.85	9	423	2.13	0.43 (0.11, 1.66)
Hepatitis C							
No	162	6022	2.69	92	6174	1.49	0.63 (0.46, 0.86) **
Yes	17	366	4.65	9	387	2.33	1.00 (0.20, 4.96)
Peripheral vessel disease							
No	177	6312	2.80	98	6493	1.51	0.58 (0.43, 0.77) ***
Yes	2	75	2.65	3	68	4.42	-
Follow-up period, years							
≤1	31	1248	2.48	34	1251	2.72	1.10 (0.67, 1.78)
>1, ≤2	27	1127	2.40	16	1130	1.42	0.64 (0.34, 1.20)
>2, ≤3	34	966	3.52	11	971	1.13	0.31 (0.14, 0.68) **
>3, ≤4	16	802	2.00	11	807	1.36	0.54 (0.22, 1.35)
>4, ≤5	16	653	2.45	10	673	1.49	0.60 (0.22, 1.65)
>5	55	1591	3.46	19	1728	1.10	0.23 (0.10, 0.56) **

Rate #, incidence rate per 100 person-years; HR, hazard ratio. * *p* < 0.05; ** *p* < 0.01; *** *p* < 0.001.

**Table 4:** Incidence and hazard ratios of mortality among patients receiving a kidney transplant with varying pretransplant dialysis durations.

Pre-Transplant Dialysis Duration (Years)	N	Event	PY	Rate #	cHR (95% CI)	aHR † (95% CI)
0–1 (reference)	101	5	468	1.07	1 (Reference)	1 (Reference)
1–3	325	22	1948	1.13	1.10 (0.42, 2.92)	1.07 (0.40, 2.83)
3–5	318	29	1727	1.68	1.62 (0.63, 4.18)	1.39 (0.53, 3.62)
5–7	252	21	1293	1.62	1.54 (0.58, 4.09)	1.41 (0.53, 3.77)
>7	280	24	1124	2.13	1.94 (0.74, 5.08)	1.65 (0.62, 4.36)

Rate #, incidence rate per 1000 person-years. cHR, crude hazard ratio; aHR, adjusted hazard ratio. aHR †: multivariable analysis including age, gender, hypertension, hyperlipidemia, diabetes, coronary artery disease, atrial fibrillation, heart disease, hepatitis B, hepatitis C, and peripheral vessel disease.

**Table 5:** Comparison using different propensity score methods for sensitivity analysis.

Variable	HR (95% CI)
Mortality	
Stratification adjusted on the propensity score 10 strata	0.54 (0.43, 0.69) ***
Inverse probability of treatment weights	0.55 (0.46, 0.65) ***
Covariate adjustment by using the propensity score	0.54 (0.42, 0.69) ***

HR, hazard ratio. *** *p* < 0.001.

## References

[1] Jager K.J., Fraser S.D.S. (2017). The ascending rank of chronic kidney disease in the global burden of disease study. Nephrol. Dial. Transpl..

[2] Schnuelle P., Lorenz D., Trede M., Van Der Woude F.J. (1998). Impact of renal cadaveric transplantation on survival in end-stage renal failure: Evidence for reduced mortality risk compared with hemodialysis during long-term follow-up. J. Am. Soc. Nephrol..

[3] Pascual J., Zamora J., Pirsch J.D. (2008). A systematic review of kidney transplantation from expanded criteria donors. Am. J. Kidney Dis..

[4] Merion R.M., Ashby V.B., Wolfe R.A., Distant D.A., Hulbert-Shearon T.E., Metzger R.A., Ojo A.O., Port F.K. (2005). Deceased-donor characteristics and the survival benefit of kidney transplantation. JAMA.

[5] Cohen J.B., Eddinger K.C., Locke J.E., Forde K.A., Reese P.P., Sawinski D.L. (2017). Survival Benefit of Transplantation with a Deceased Diabetic Donor Kidney Compared with Remaining on the Waitlist. Clin. J. Am. Soc. Nephrol..

[6] Jay C.L., Washburn K., Dean P.G., Helmick R.A., Pugh J.A., Stegall M.D. (2017). Survival Benefit in Older Patients Associated with Earlier Transplant with High KDPI Kidneys. Transplantation.

[7] Teixeira J.P., Combs S.A., Teitelbaum I. (2015). Peritoneal dialysis: Update on patient survival. Clin. Nephrol..

[8] Rigoni M., Torri E., Nollo G., Zarantonello D., Laudon A., Sottini L., Guarrera G.M., Brunori G. (2017). Survival and time-to-transplantation of peritoneal dialysis versus hemodialysis for end-stage renal disease patients: Competing-risks regression model in a single Italian center experience. J. Nephrol..

[9] Wu M.J., Lo Y.C., Lan J.L., Yu T.M., Shu K.H., Chen D.Y., Ho H.C., Lin C.H., Chang S.N. (2014). Outcome of lupus nephritis after entering into end-stage renal disease and comparison between different treatment modalities: A nationwide population-based cohort study in Taiwan. Transpl. Proc..

[10] Tennankore K.K., Kim S.J., Baer H.J., Chan C.T. (2014). Survival and hospitalization for intensive home hemodialysis compared with kidney transplantation. J. Am. Soc. Nephrol..

[11] Storey B.C., Staplin N., Harper C.H., Haynes R., Winearls C.G., Goldacre R., Emberson J.R., Goldacre M.J., Baigent C., Landray M.J. (2018). Declining comorbidity-adjusted mortality rates in English patients receiving maintenance renal replacement therapy. Kidney Int..

[12] Wetmore J.B., Gilbertson D.T., Liu J., Collins A.J. (2016). Improving Outcomes in Patients Receiving Dialysis: The Peer Kidney Care Initiative. Clin. J. Am. Soc. Nephrol..

[13] van Walraven C., Manuel D.G., Knoll G. (2014). Survival trends in ESRD patients compared with the general population in the United States. Am. J. Kidney Dis..

[14] Pippias M., Jager K.J., Kramer A., Leivestad T., Sanchez M.B., Caskey F.J., Collart F., Couchoud C., Dekker F.W., Finne P. (2016). The changing trends and outcomes in renal replacement therapy: Data from the ERA-EDTA Registry. Nephrol. Dial. Transpl..

[15] Roberts M.A., Polkinghorne K.R., McDonald S.P., Ierino F.L. (2011). Secular trends in cardiovascular mortality rates of patients receiving dialysis compared with the general population. Am. J. Kidney Dis..

[16] Deb S., Austin P.C., Tu J.V., Ko D.T., Mazer C.D., Kiss A., Fremes S.E. (2016). A Review of Propensity-Score Methods and Their Use in Cardiovascular Research. Can. J. Cardiol..

[17] Yu T.M., Chuang Y.W., Yu M.C., Chen C.H., Yang C.K., Huang S.T., Lin C.L., Shu K.H., Kao C.H. (2016). Risk of cancer in patients with polycystic kidney disease: A propensity-score matched analysis of a nationwide, population-based cohort study. Lancet Oncol..

[18] Huang C.C., Cheng K.F., Wu H.D. (2008). Survival analysis: Comparing peritoneal dialysis and hemodialysis in Taiwan. Perit. Dial. Int..

[19] Wu P.H., Lin Y.T., Lee T.C., Lin M.Y., Kuo M.C., Chiu Y.W., Hwang S.J., Chen H.C. (2013). Predicting mortality of incident dialysis patients in Taiwan—A longitudinal population-based study. PLoS ONE.

[20] Silbernagel G., Genser B., Drechsler C., Scharnagl H., Grammer T.B., Stojakovic T., Krane V., Ritz E., Wanner C., März W. (2015). HDL cholesterol, apolipoproteins, and cardiovascular risk in hemodialysis patients. J. Am. Soc. Nephrol..

[21] Neale J., Smith A.C. (2015). Cardiovascular risk factors following renal transplant. World J. Transpl..

[22] Ramphul R., Fernandez M., Firoozi S., Kaski J.C., Sharma R., Banerjee D. (2018). Assessing cardiovascular risk in chronic kidney disease patients prior to kidney transplantation: Clinical usefulness of a standardised cardiovascular assessment protocol. BMC Nephrol..

[23] Helantera I., Salmela K., Kyllonen L., Koskinen P., Gronhagen-Riska C., Finne P. (2014). Pretransplant dialysis duration and risk of death after kidney transplantation in the current era. Transplantation.

[24] Haller M.C., Kainz A., Baer H., Oberbauer R. (2017). Dialysis Vintage and Outcomes after Kidney Transplantation: A Retrospective Cohort Study. Clin. J. Am. Soc. Nephrol..

[25] Mokos I., Basic-Jukic N., Kastelan Z., Kes P., Pasini J. (2010). Influence of long-term dialysis treatment on operative complications after renal transplantation. Transpl. Proc..

[26] Batabyal P., Chapman J.R., Wong G., Craig J.C., Tong A. (2012). Clinical practice guidelines on wait-listing for kidney transplantation: Consistent and equitable?. Transplantation.

[27] Johnson D.W., Herzig K., Purdie D., Brown A.M., Rigby R.J., Nicol D.L., Hawley C.M. (2000). A comparison of the effects of dialysis and renal transplantation on the survival of older uremic patients. Transplantation.

[28] Knoll G., Cockfield S., Blydt-Hansen T., Baran D., Kiberd B., Landsberg D., Rush D., Cole E. (2005). Kidney Transplant Working Group of the Canadian Society of Transplantation. Canadian Society of Transplantation consensus guidelines on eligibility for kidney transplantation. CMAJ.

[29] Grams M.E., Massie A.B., Schold J.D., Chen B.P., Segev D.L. (2013). Trends in the inactive kidney transplant waitlist and implications for candidate survival. Am. J. Transpl..

[30] Grams M.E., Massie A.B., Coresh J., Segev D.L. (2011). Trends in the timing of pre-emptive kidney transplantation. J. Am. Soc. Nephrol..

